# *Cyclamen persicum* Mill. and *Leontice leontopetalum* L., common vernacular names – and their relation to washing, incense and the unexplained ‘Jordan Dome’

**DOI:** 10.1186/s13002-026-00890-9

**Published:** 2026-03-29

**Authors:** Amots Dafni, Salah ‘Aql Khatib, Barbra Böck

**Affiliations:** 1 Department of Evolutionary and Environmental Biology and Institute of Evolution, University of Haifa, Haifa, Israel; 2 Mghar, 20128 Israel; 3 Instituto de Lenguas y Culturas del Mediterráneo y Oriente Próximo, CSIC, Madrid, Spain

**Keywords:** *Cyclamen persicum*, *Leontice leontopetalum*, “Jordan Dome”, Incense plants, Washing plants, Identification of Semitic vernacular names

## Abstract

**Background:**

Vernacular plant names reflect cultural perceptions, historical interactions, and practical uses. The names of *Cyclamen persicum* and *Leontice leontopetalum* exhibit complex historical transformations across the Middle East.

**Methods:**

We examined linguistic and historical sources alongside botanical, ethnobotanical, and phytochemical data to trace the origins, meanings, and diffusion of the vernacular names of these two species.

**Results:**

Comparative analysis revealed overlapping semantic fields related to washing textiles and incense use, which contributed to the convergence of vernacular names in different languages and regions. Historical and philological evidence supports the identification of the Mishnaic *‘Kippat ha-Yarden*’ (‘Jordan Dome’) with *Leontice leontopetalum*, as hypothesized by Immanuel Löw.

**Conclusions:**

Integrating linguistic, historical, botanical, and ethnobotanical data provides new insights into the evolution of vernacular plant nomenclature and contributes to the identification of problematic plant names in ancient sources.

## Introduction

Plant names are the essence of a people’s bond to their land and reflect the interaction between human culture and local nature opening a window into the linguistic, cultural, and intellectual development of the societies from antiquity to the modern era. Far from being a simple matter of popular vocabulary, vernacular naming practices reflect shifting patterns of language contact, scholarly authority, translation, and identity formation. How vernacular names were transformed, contexts resemanticized and how lexical items were coined was an important issue which was discussed especially among Jewish Botanists at the beginning of the 20th century.

To understand the intensified interest in philology and historical linguistics concerning plant names it is worthwhile quoting the observations of the following three scholars:

Aharonson [1] observed: “Vernacular plant names were never given to plants by any nation at random… their origin lies in some ancient legend… The way these names were fixed sheds light on the process… usually we find in their folk-name a deep symbolism… Vernacular plant names have their own plant lore, most often based on folklore… The original plant names were frequently corrupted, and we can often reconstruct their meaning only through conjecture and interpretation.”

Sapir [2] addressed a similar issue: “The language of plants is, to a large extent, the language of the people of the land, and it is generally preserved in its primitive popular form. The people, especially those dwelling in the fields and shepherds, create plant names according to the images revealed to them, the properties they recognize, the virtues they find in them, or chance associations—sometimes intentionally and sometimes incidentally. Thus, members of one people, within their unique language, may nonetheless give different names to the same plant depending on their locality, as people in different places perceive and emphasize different qualities. Conversely, different peoples inhabiting the same land, though speaking different but related languages, may often call the same plant by identical or nearly identical names.”

Tchernichovsky [3] also discussed this phenomenon: “…the names of flowers are by no means primeval; they are liable to change. There are names being formed before our eyes right now, flowers that as yet have no name among the people, which are about to acquire one. There are flower names preserved only in manuscripts… with no longer any trace in the living language. From this we can see the multiplicity of names applied to one flower. An ancient name may vanish in one district and survive in another. In some cases, a new name is given when a property previously unknown is discovered; attention is drawn to such a plant and it is then named, whereas earlier it bore no name. Conversely, when belief in its virtues ceases and it falls out of use, its very name is forgotten… Clearly then, flower names are not permanent; some arise and some vanish and wither away, like the flowers themselves.”

The study of the history of vernacular names in Hebrew and in Jewish sources is particular demanding because of the particular evolution of the vernacular terminology and serves, thus, to exemplify the process of name-giving in general. Few names for the flora are attested in the Hebrew Bible; though they convey local ecological and cultural knowledge, there is naturally lexical uncertainty about the identification of the plant names. Many terms became opaque in late antiquity prompting interpretive efforts in the Aramaic Targumim and later rabbinic literature. In a gradual process starting in the 6th century BCE Hebrew was gradually replaced by Aramaic. Vernacular equivalences of plant names were supplied through translation, glosses, and commentaries, creating layered systems of naming in which Hebrew, Aramaic, Arabic, and later European languages interacted. During the Medieval Ages, especially in the Islamic world, Jewish scholars writing in Judeo-Arabic engaged deeply with Greco-Arabic science and medicine. Figures such as Moses Maimonides frequently correlated Hebrew terms with Arabic scientific nomenclature, attempting to stabilize identifications of plants and medical substances mentioned in classical Jewish sources.

The challenge of identifying plants mentioned in Jewish sources after a two-thousand-year separation from the Land of Israel becomes thus evident. The most influential authority in this field is Immanuel Löw, whose monumental *Die Flora der Juden* [4] remains foundational. In this comprehensive work, Löw identified most plants in Jewish sources, drawing on his extensive knowledge of ancient and modern languages, his deep expertise in Jewish and general literature, and his systematic philological methodology.

Subsequent Israeli scholars, including Ephraim and Hannah Ha-Reuveni, Uriyah Feldman, Baruch Tchizik, Michael Zohary, Yehuda Feliks, and, in more recent decades, Mordechai Kislev and Zohar Amar, have further developed and expanded Löw’s pioneering foundations. Contemporary research has increasingly adopted an integrative, multidisciplinary approach that combines historical and philological sources with modern etymological analyses—particularly those pertaining to Mesopotamian traditions—together with botanical (phytogeographical, morphological, and ecological) and ethnobotanical data, as well as current phytochemical investigations. These complementary methodologies serve as critical tools for elucidating problematic plant identifications in Jewish textual sources. Illustrative examples include recent proposals identifying the biblical ʾātād with species of *Astragalus* [5]; “*Kippat ha-Merḥaṣ*” with *Hyoscyamus niger *[6],“Dove’s Dung” with *Prosopis farcta* [7], and biblical *ṣori* either with *Styrax officinalis* resin [8] or with *Pistacia atlantica* resin [9].

In the present article, following a multidisciplinary approach, we examine the historical development of the names of *Cyclamen* and *Leontice*, tracing the associations between them as expressed through shared uses and vernacular terminology. The choice is due to philological, ethnobotanical, historical, and cultural grounds. Both plants occupy a distinctive position at the intersection of classical texts, medieval pharmacology, vernacular plant nomenclature, and lived ecological knowledge in the Eastern Mediterranean region. The two species are embedded in ritual, medicinal, and symbolic frameworks; they are native to the Eastern Mediterranean basin, a region which is characterized by intense cultural layering. Their survival across millennia makes them particularly apt indicators of the entanglement between ecological persistence, cultural memory and naming practices.

We also provide supporting evidence for Immanuel Löw’s hypothesis that the Mishnaic *‘Kippat ha-Yarden’* (‘Jordan’s Dome’) should be identified with *Leontice leontopetalum L*.- and a proposal that has not yet been fully accepted in the scholarly literature.

This contribution aims at reconstructing the historical, ethnobotanical, ritual, and etymological origins of the vernacular names of *Cyclamen persicum* and *Leontice leontopetalum*. So far, no study has been carried how both taxa are embedded in the Greco-Roman textual tradition and how its tuber, purgative properties, and ritual associations shaped later Byzantine, Syriac, Arabic, and Hebrew pharmacological literature. Taking into account their use for washing textiles and as incense throughout the Mediterranean region the study seeks to shed light on how Mediterranean societies moved from descriptive, symbolic, and use-based naming systems to standardized botanical nomenclature. By tracing the textual attestations, vernacular names, medicinal uses, and modern taxonomic identities broader processes are illuminated such as the transmission of knowledge between Greek, Byzantine, Jewish, Islamic, and European traditions.

## Methods

Following the methodological principles proposed by Sorokin [10] and, in part, by Lardos et al. [11] regarding the identification of plant names in historical sources, we adapt and refine their approach within the following analytical framework:

1. **Source Survey** – A comprehensive review of the primary and secondary sources in which names considered to be associated with *Cyclamen* and/or *Leontopetalum* appear in the literature.
2. **Philological Analysis** – Close examination of the original texts, with particular attention to the contextual use, semantic range, and functional meaning of the relevant plant names.
3. **Lexical and Etymological Study** – Comparative linguistic analysis of the names across closely related Semitic languages, aiming to clarify their roots, semantic shifts, and patterns of borrowing or diffusion.
4. **Medical and Ethnobotanical Documentation** – Survey of medical treatises in which these taxa are mentioned, accompanied by a critical review of available ethnobotanical data.
5. **Botanical Correlation** – Evaluation of the morphological and ecological characteristics of the surveyed species in order to assess the plausibility of proposed identifications.
6. **Phytochemical Considerations** – Consideration of modern phytochemical data as an interpretive tool for assessing the feasibility of the attributed traditional uses.

Finally, all lines of evidence were synthesized in order to integrate the philological, ethnobotanical, botanical, and chemical data into a comprehensive interpretative framework. This multidisciplinary approach was designed to elucidate the etymology, semantic development, diffusion, and historical trajectories of the vernacular names attributed to the two species.

## Results and discussion

### The scientific name of the common cyclamen

The genus *Cyclamen* is currently classified in the subfamily Myrsinoideae, within the family Primulaceae. It comprises 25 species, most of which are distributed around the Mediterranean Basin [12].

The scientific name *Cyclamen* derives from the Greek word κύκλος (*kýklos*), meaning ‘circle’ or ‘ring,’ referring to the curled flower stalk, the round shape of the leaves, and, according to some interpretations, the form of the tuber [13]. According to Sirianni [14] the term derives from Greek κύκλα (*kykla*, the collective form meaning ‘wheel’), where the semantic core is circular movement. Multiple circular features observed: The helical arrangement of petals during flowering. The spiral movement – ‘open’ spiral of the flower bud and ‘closed’ spiral of the fruit-root. While acknowledging the traditional ‘round root’ etymology, the author advocates for understanding the name as referring to the plant’s circular/spiral growth cycle rather than merely its bulb shape, suggesting this reflects sophisticated ancient botanical observation.

The Greek name *kyklaminos* (κυκλάµινος) appears, for the first time, in the work of the Greek botanist Theophrastus (371–287 BCE), who likely coined the term [15], and in several works ascribed to Hippocrates of Cos (ca. 460 – ca. 370 BCE) such as the gynecological treatises [16]. From this point, the name spread into many European languages.

The binomial name *C. persicum* Mill., meaning ‘Persian *Cyclamen*,’ was published in 1768 by Philip Miller [17], who believed that the plant originated in Persia. However, *Cyclamen persicum* had already been cultivated in European gardens for over a century under various names. The earliest probable evidence dates to the late 16th century, when Carolus Clusius received tubers from Constantinople in the 1590’s, although their identification remains uncertain. More concrete records appear in the 17th century: by 1665, the Royal Botanical Garden in Paris possessed several *Cyclamen* varieties, including one referred to as ‘Persicum dictum’, reflecting the early assumption of a Persian origin [18].

The common *C. persicum* does not occur naturally in Iran. In the wild, it is found in the eastern Mediterranean and northwestern Africa, including Greece, Crete, Cyprus, Turkey, Syria–Lebanon, Israel–Palestine, Jordan, Algeria, and Tunisia. Many of today’s horticultural cultivars originate from these wild populations [19]. Around the Mediterranean, *Cyclamen* has numerous vernacular names, but the equivalent to ‘Persian *Cyclamen*’ occurs mainly in Catalan, Portuguese, and French [20].

### The origin of the name leontice leontopetalum

*Leontice leontopetalum* is an Irano–Turanian–Mediterranean species, distributed from Iran in the east to the Balkans in the north and Morocco in the west [21]. Notably, the species does not occur in Western Europe, a fact of particular importance for the discussion of its nomenclatural history. Linnaeus described the species under the valid scientific name *L. leontopetalum* on the basis of a Middle Eastern specimen [22].

The genus name *Leontice* derives from the Greek λέων (*léōn*, ‘lion’) combined with the suffix **-**ικος (*-ikos*), meaning ‘belonging to’. The species epithet *leontopetalum* is a compound of πέταλον (*pétalon*, ‘petal’ or ‘leaf’, usually ‘petal’) and λέων (*léōn*). According to Dioscorides (1st century CE), the name refers to the leaf shape, which was said to resemble a lion’s paw [23].

The Greek name migrated eastward. The earliest Arabic source to associate the element ‘lion’ (*asad*) with the name *Leontice* is attributed to Abū Ḥanīfa al-Dīnawarī (9th century), who uses the expression ‘leg of the lion’ (جل الأسد *rajal al-asad*), justified by the resemblance of the leaves to a lion’s paw [24]. Bar-Bahlul, a 10th -century Syriac lexicographer, similarly notes that the plant has a tuber and leaves resembling a lion’s paw [25].

### On the confusion between cyclamen and leontice

The earliest evidence of confusion between *Cyclamen* and *Leontice* appears in the writings of Avicenna (Ibn Sina, 980–1037 CE, who lived and worked in Persia. Natan Ha’Meati [26], translating Avicenna’s Canon into Hebrew at the 15th century, distinguishes between *Artanita* and *Bakhur Maryam* implying separate identities for these terms in modern translations of the Canon, *Artanita* is equated with *Cyclamen*, and *Bakhur Maryam* with *Leontice* [27].

Ibn Waḥshīyah (9th − 10th centuries) identifies *Artanita* as the tuber of *Cyclamen* [28]. Al-Qazwīnī (1201–1283 CE) later writes: “*Shagger Maryam* is *Bakhur Maryam*, it is thorny [sic.], and it is the root of *Artanita*” [29].

The Andalusian Arabic dictionary [30] defines *ātranīpā* as *Cyclamen europaeum* (‘European Cyclamen’, which is recognized today as synonym of *Cyclamen purpurascens* Mill. *subsp*. *purpurascens*), meaning “incense-like.” On p. 464, *Kaff al-Asad* (‘Lion’s Paw’) is listed for *Cyclamen*—a name that in fact belongs to *Leontice*.

Maimonides, in his *Explanation of the Names of Medicaments* [31], refers to both *Cyclamen* and *Leontice* multiple times: “*Saggarat Maryam*, which is called *Qaklaminos* [the Greek name for *Cyclamen*] is not at all identical to *Bakhur Maryam* as many physicians suppose” (#64). The editor, Ziesman Muntner, comments that the two are frequently confused and that *Artanita* is referred to inconsistently. Natan Ha-Me’ati (1280–1340 CE, Italy), in his Hebrew translation of Avicenna’s *Canon* [26], renders *Shaggarat Maryam* as ‘Tree of Mary’, and the glossary compiler identifies this with *Cyclamen* [32].

In the same work, Maimonides writes:*“Artanita*, already described, is the root of one of two species of *Arum* (*Arum*), also called ‘monkey bread’” [31]. He continues regarding *Bakhur Maryam* (‘Mary’s Incense’): “Later experts of plants applied this term to the roots of *Adryon* (*Adrion*, Arabic: آذريون), known in Spain as *Zahabiya* (‘golden,’ in Arabic) from its golden flowers, and in Spanish as *Jerjeritya*. When the flowers fall, they form something like a hand, hence the name ‘Lion’s Paw’ (كف الأسد) — Greek *Kyklaminos*. This is entirely not the Tree of Mary (*Shaggarat Maryam*), which is a different plant, while *Bakhur Maryam* is *Cyclamen*.”

The editor of Maimonides [32] lists eight possible identifications for *Shaggarat Maryam*: *Bakhur Maryam*, *Tsarda*, *Ashlag*, *Kippat ha-Yarden*, *Artanit*, *Cyclamen*, and *Calendula officinalis*.

From this section one may conclude that:

1. Maimonides clearly distinguishes *Bakhur Maryam* from *Shaggarat Maryam*.
2. *Bakhur Maryam* refers to *Cyclamen*, based on its attested Greek name from pre-medieval sources.
3. There is no connection between *Arum* and *artanita*; ‘Monkey Bread’ is a familiar vernacular name for *Cyclamen*.
4. *Adrion*, according to Maimonides, is a name for *Leontice*; the description of golden flowers matches this identification.
5. The name ‘Lion’s Paw’ is well attested for *Leontice*. Its association with Greek *Kyklaminos* (*Cyclamen*) is a later development. The ‘Tree of Mary’ generally refers to *Cyclamen*.

Al-Ghafīqī (12th century, al-Andalus), when discussing *Cyclamen*, cites Idrīsī, who lists the names *Bakhūr Maryam*, *Al-ʾAdrion*, and *al-Warka* in Syria, with the root called *Artanīta* and the common name *Hubz al-Qurūd* (‘Monkey Bread’). The editor’s note the following synonyms: *Kyklaminos*, *Tuber Terrae* (‘Earth Bread’), in Syriac *Artanīta*, and in Arabic: *Bakhūr Maryam*, *Shaggarat Maryam*, *Hubz al-Qurūd* (‘Monkey Bread’), *Hubz al-Mashayikh* (‘Bread of the Sheikhs’), and *Hubz al-Khanāzīr* (‘Pig’s Bread’) [33].

In European literature, the name ‘Pig’s Bread’ for *Cyclamen* appears as early as in a 14th -century manuscript [34, 39] and later entered Hebrew and Arabic texts. Seldino di Ascoli, a 15th -century’ Jewish pharmacist in Italy, in his *Pharmacists’ Book* on methods of drug preparation, wrote: “Artanita = Atanita or ‘Bread of Tūt’ [35].

### Identification of Shaggarat Maryam

Already in Ibn al-Bayṭār’s work, *Shaggarat Maryam* refers to several species, some of which cannot be identified with certainty [36]. Numerous manuscripts of Ibn al-Bayṭār’s book exist, with many variant readings [37]. This variation has led to multiple identifications. For Example, Leclerc’s French translation [38] and Sontheimer’s German version [39] are based on different manuscripts which may explain the differences between them.

Ducros [40], reviewing the early 20th -century popular herbal market in Cairo, under the entry *Artanīta*, notes that European *Cyclamen* (*C. europaeum*, see above), which is not native to the Middle East, was used by ancient pharmacists under the name *Artanīta*, meaning *Cyclamen* or ‘Pig’s Bread’, which Arabs translated as *Hubz al-Khanāzīr*. It is also known as *Paklaminon* (a distorted Greek *Kyklaminon*), *Karn al-ʿAzal* (‘Deer Horn’), *Bakhūr Maryam* (‘Mary’s Incense’), and *Hubz al-Mashayikh* (‘Bread of the Sheikhs’). The latter two names are mostly Syrian and North African (Tripolitania, Tunisia, Algeria). Sometimes *Cyclamen* is also called *Shaggarat Maryam* (‘Tree of Mary’), a name shared by several plants including *Parthenium* [= *Tanacetum parthenium* L.] the ‘Shrub of Abraham’ (*Vitex agnus-castus* L.), and *Cachrys* [hic!], and is therefore not unique to *Cyclamen* [40].

According to Mundhir Bayṭār (14th century, Egypt), *Shaggarat Maryam* = *Artanīta* = *Bakhūr Maryam* = Tree of Mary = *Kaff Maryam* = *Skuka* and *Duwīk al-Jabal* (the latter two being Arabic folk names for *Cyclamen*). The name *Bakhūr Maryam* was also applied to other plants. The editor notes that *Artanīta* is an Aramaic word referring to an herbaceous perennial of the Primulaceae family with a beautiful flower, that is, *Cyclamen* [41].

This section concludes with the observation that the confusion between *Cyclamen* and *Leontice*, which originated in the 10th century in the East, extended to the writings of Maimonides and subsequently spread westward.

### Cyclamen and leontice as washing plants

One of *Cyclamen*’s Arabic names is *Ṣābūn al-Rāʿī* (صابون الراعي, Shepherd’s Soap). Traditionally, shepherds rubbed the sliced tuber on a wet stain and then washed the fabric to remove it. A legend recounts that Moses already knew this practice, hence the additional nickname *Ṣābūnit Sayyidnā Mūsā* (صابونة سيدنا موسى, Our Master Moses’s Soap). This name does not appear in classical sources and has so far been found only in modern literature from Israel [42]. In Turkey, Greek *Cyclamen* (*C. coum* Mill.) is used similarly [43].

*Cyclamen* tubers contain saponins [44, 45], known for their foaming and antibacterial properties [44, 46], making the plant suitable for use as soap. Saponins reduce the surface tension of water, enhancing its penetration into fabric fibers to remove soil effectively. They also create stable foam and emulsions with oils, facilitating stain removal [47].

The tuber of *Leontice* is round and fleshy, resembling that of *Cyclamen*, and also contains saponins [48]. Some sources claim that *Leontice* was used as soap already in Assyria, although no reference substantiates this [49, 50]. Ibn Sina noted that *Artanita* (= *Bakhur Maryam* = *Cyclamen*) was used for washing wool garments [25].

It has been suggested that *Leontice* and *Cyclamen* were used in ancient Mesopotamia as soap plants, viz. as purifying agent in rituals and as medicinal ingredients [50]. The plants were thought to be known under the names *tullal* (*Leontice*) and dil.bat. (*Cyclamen*). However, the identification is based on fallacious etymology. The name *tullal was related to the root *ʾll* ‘to cleanse purify’, hence the meaning of the name ‘thou-shall-wash (plant)’ [50]; the reading of the cuneiform signs is erroneous and should be corrected into *tulla*, a word of unknown linguistic [41]. The same holds true for the term *dil.bat* which was interpreted as ‘Venus flower’ and associated with *Bakhur Maryam* [50]. The cuneiform signs should be read *in.úš*; also this name is of unknown linguistic origin.

### Cyclamen and leontice as incense plants

*Cyclamen* is called in Arabic *Bakhur Maryam* (بخور مريم, Mary’s Incense). This name also appears in Persian and Turkish (*buhurmeryem*). It applies equally to *Leontice* in early and later literature. The earliest Arabic source calling *Cyclamen Bakhur Maryam* is al-Razi (865–925), who lived in Iraq and Persia [52]. Ibn Sina (10th − 11th century) names *Cyclamen* both *Bakhur Maryam* [25] and *Artanita* (p. 320). The Cairo Genizah (10th − 13th centuries) also lists *Bakhur Maryam* as a synonym for both *Cyclamen* and *Leontice* [53]. Zrakhiya ben Yitzhak ben Shaltiel Hen, active in 14th -century Spain and Rome, translated the second volume of Ibn Sina’s Canon into Hebrew, rendering *Shaggarat Maryam* as *Artanita* or ‘Mary’s Herb’, amended by the editor as ‘Mary’s Smoke’ [54], possibly alluding to incense use.

The name *Bakhur Maryam* for *Leontice* first appears in Arabic literature in 1083, in a translation of Dioscorides’s Materia Medica into Arabic at Samarkand, Uzbekistan [55]. It is noteworthy that the introduction of incense into Christian ritual is first seen in the Syriac church in the late fourth century [56], while the earliest records of ‘Mary’s Incense’ are from the 10th century.

Evidence for *Cyclamen*’s use as incense is mainly linguistic and symbolic. No direct written testimony confirms that *Cyclamen* or *Leontice* tubers were actually burned as incense. Gustav Hegi [57] notes and reports that dried *Cyclamen* tubers were occasionally burned as incense in monasteries, citing the name ‘Mary’s Incense’, but without written proof.

In Persian, *Leontice* tuber is called Āzārbūyeh (آذربویه), derived from Āzar (آذر, fire) and Būye (بویه, smell or aroma), hence meaning ‘smell of fire’, i.e., incense for burning [58]. The earliest explicit mention of preparing incense from *Leontice* appears in Bar-Bahlul (10th century): “*Bakhur Maryam* ( ʿAlṭīnā, i.e., *Leontice*) as in Europe, has a very large tuber from which incense (*qaṭar*) is made for its virtue of long life” [25].

An Andalusian Arabic source notes that *Artanita* in the East refers solely to *Leontice*. When Arabs reached Spain and did not find *Leontice*, they substituted the local *Cyclamen* (*C. europaeum*, see above), also calling it *Artanita*. In Morocco, due to the scarcity of *Cyclamen*, another local plant was used as incense, though the part used is unspecified [58].

### The possible identification of ‘Jordan Dome’

Immanuel Löw proposed in *‘Aramaic Names of Plants* that  (ʿ*arṭānīṯā*) is a corruption of  (ʿ*aṭrānīṯā*), both deriving from  (ʿ*aṭrānā*), meaning “cedar resin, tar,” whose Arabic cognate is *qaṭrān* (قَطْرَان).

[59]. The Syriac ʿaṭrānā shows a semantic connection to incense. The Arabic and Syrian equivalents ʿ*arṭanīṯā* (, *Artanita*) confirm a Semitic root associated with burning and incense, paralleling Hebrew *ketar* and *lehaqṭir*.

Kogan and Krebernik [60] note that the related verbal root ḳṭr, ‘to fumigate’, is well attested across Semitic languages, including Hebrew, Mandaic, Arabic, Sabaic, and Geʿez. The meaning ‘smoke’ goes back to Akkadian influence (for *qutru* ‘smoke, fume’ see [61, 62]; for *qatāru* [qutturu] ‘to incense, fumigate, smoke’ see [61, 62]. Akkadian has formed from this root the plant names *qutru* and *qutratu*, which are both of unknown botanical identification.

Löw, in *‘Die Flora der Juden*, postulated that the incense plant *Artanita* was originally *aṭrānīta*, later altered to a name of unclear etymology (4, I:289).

The T–R exchange in *Atran/Artan* follows established patterns of consonant metathesis in Semitic languages, where liquids like/r/frequently interchange positions with adjacent consonants. Such reordering is well-documented in Arabic dialects, Hebrew, and Aramaic [64, 65]. Modern studies of Syrian Arabic confirm extensive T–R exchange patterns consistent with this historical process [65, 67].

Löw further suggested that in the name *Kippat ha*-*Yarden* (‘Jordan’s Dome’), a scribal error occurred, and it should read *Kippat ha-ʿArtan*, meaning ‘Dome of the Artan’, referring to a plant name [4, III:353; IV:100]. *Kippat ha-Yarden* is mentioned as one of the incenses burned in the Temple: “One raises smoke, a herb causing smoke of pleasant incense… Rabbi Natan says: also *Kippat ha-Yarden*, or whatever it may be” (Mishna, Keritut 6a).

Krupnik and Silbermann [68] identify *‘Kippat ha-Yarden*’ as *Cyclamen*. Felix [69], following Löw, suggests it is probably *Cyclamen* while Amar [70] finds no confirming evidence for any suggestion. Monferrer-Sala [71] suggests that the Arabic word *bakhur* translates the Greek *líbanon* (λίβανον), derived from the Hebrew *levona* (‘frankincense’), corresponding to the Aramaic-Syriac *Artanita* (Arṭanītā), i.e., *Leontice leontopetalum*.

The Spanish online dictionary traces *Artanita*—a Spanish name for *Cyclamen*—to the Arabic *artanita* of Syriac origin, meaning “resembling incense” [72]. This connection reinforces Löw’s hypothesis that *‘Kippat ha-Yarden’* means ‘Dome of the *Artan*’ (Dome of the *Leontice*). Supporting evidence comes from Payne-Smith’s Syriac Dictionary [73], which gives *Cyclamen* as  (*kpaṯ ʿarṭānā*, “Dome of the Artan”). The same source [73] lists  (kpaṯā) = *Cyclamen* and  (*kpaṯīṯā/kpaṯṯā*) = *Leontice* or *Cyclamen*. The Sureth Dictionary identifies *Cyclamen kap̄āṯ ʿarṭānā* () as “Dome of the Artan” in the Urmia dialect [74].

Both species have globular tubers, justifying the epithet ‘Dome’ (*kippa* in Syriac and Hebrew). The name *Artanita* has accompanied both for centuries, forming an etymological continuum: *aṭranita* → *artanita* → ‘Dome of the *Artan* (Jordan)’ → *aṭran* → *qatran* → *ktoreth* (incense in Hebrew).

Research on Turkish poetry mentioning *Cyclamen* (*buhurmeryem*, ‘Mary’s incense’, borrowed from Arabic) indicates that *Cyclamen* was used as incense in churches in Turkey. *Cyclamen* flowers were among the ingredients of ‘incense water’ used in Ottoman palaces in the 16th century [75, 76]. In Armenia, *Cyclamen* flower is used in preparing the Holy Muron, the sacred oil for religious ceremonies [77].

### Cyclamen–Leontice Name Interchange

Several factors account for the persistent interchange of names between these two plants. The resemblance of their globular tubers (‘domes’) likely underlies both the names *‘Jordan’s Dome’”* and *‘Dome of the Artan’.* Their similar chemical composition, particularly their shared saponin content, further contributes to this identification.

1. If both plants were used for incense, their tubers lack the aromatic volatile substances suitable for combustion. It is hypothesized that the saponins in both tubers inhibited combustion, prolonging burning. Studies show that saponins reduce burn temperature and smoke production while delaying combustion [78, 79], which may have improved incense performance.
2. In European pharmacological literature, where *Leontice* does not grow, *Artanita* was occasionally used for *Cyclamen* [80, 81], especially in homeopathic texts. No shared medicinal use explains the substitution, but the similar tubers and their use in dried form for remedies likely caused misidentification.
3. Both species served as soap plants in the Middle East and Turkey because of high saponin content. A pharmacological history book notes: “*Artanita* denotes both *Leontice* and *Cyclamen*. Both contain saponins with a bitter taste producing abundant foam. These shared chemical properties explain the common name” [82].

This issue was already discussed in the 18th century, when *Leontopetalum* was considered synonymous with *Artanita*. The source adds that Ibn Sina used Artanita, but the plant was certainly *Cyclamen*, i.e., the *Leontopetalon* of the Greeks [83], *Leontopetalon*). The confusion, however, predates and postdates Ibn Sina. In his works, synonyms for *Cyclamen* include *Shaggarat Maryam*, *Bakhur Maryam*, and *Artanita* [25:284, 319, 449).

## Conclusions

This study exemplifies the historical layering of plant vernaculars and their semantic evolution over generations. Modern names reflect only the visible trace of complex linguistic and cultural histories. Scientific names of *Cyclamen* and *Leontice* derive from Greek descriptors of floral or foliar morphology, whereas the ancient name *Artanita* predates these and became entwined with both due to morphological and chemical resemblance.

The confusion of *Cyclamen* and *Leontice* in Arabic, Hebrew, Syriac, and Aramaic sources revolves around shared names—particularly *Bakhur Maryam* and *Shaggarat Maryam*—and overlapping uses. Multiple manuscript variants further complicate historical identification. Integrating linguistic, ethnobotanical, and botanical data enhances confidence in interpreting these names and their meanings.

Indirect evidence supports Löw’s hypothesis that the *Kippat ha-Yarden* (‘Jordan’s Dome’) mentioned among Temple incense ingredients refers to *Leontice leontopetalum*, whose saponins may have prolonged incense burning. The morphological similarity and shared functional uses of *Cyclamen* and *Leontice* likely generated centuries of overlap in naming and usage, a phenomenon that persists in the Middle East to this day.

## Data Availability

No datasets were generated or analysed during the current study.
